# Advanced small cell lung cancer with severe hyponatremia: a case report and literature review

**DOI:** 10.3389/fonc.2025.1558986

**Published:** 2025-09-05

**Authors:** Xu Li, Tongtong Xu, Xian Jian

**Affiliations:** ^1^ Department of Thoracic Surgery, Shengjing Hospital of China Medical University, Shenyang, China; ^2^ Department of Obstetrics and Gynecology, Shengjing Hospital of China Medical University, Shenyang, China

**Keywords:** small cell lung cancer, hyponatremia, syndrome of inappropriate antidiuretic hormone secretion, neuroendocrine tumor, lung cancer

## Abstract

Small cell lung cancer (SCLC) is a rare pathological type of lung cancer, frequently associated with neuroendocrine symptoms such as hyponatremia. This article presents a case involving a 59-year-old male patient admitted to the hospital with neurological symptoms and severe hyponatremia. He was diagnosed with SCLC accompanied by syndrome of inappropriate antidiuretic hormone secretion (SIADH) upon admission. Following oral and intravenous sodium supplementation, along with the administration of tolvaptan, the patient’s serum sodium levels increased. However, upon initiating chemotherapy, the patient’s hyponatremia worsened, leading to seizures and the need for ventilator support therapy. Despite normalization of serum sodium levels, the patient’s symptoms did not improve. Ultimately, due to the severity of the patient’s condition, the family elected to discontinue further medical intervention and proceeded with hospital discharge. Thus, in clinical practice, when encountering unexplained refractory hyponatremia with lung lesions, clinicians should consider the possibility of lung cancer with SIADH to ensure timely and precise treatment.

## Introduction

Small cell lung cancer (SCLC) is a distinct type of lung cancer known for its rapid growth, early metastasis, ectopic hormone secretion, and extrathoracic manifestations, with a notably poor prognosis. The release of endocrine-active substances by cancer cells often results in paraneoplastic syndromes, one of which is the syndrome of inappropriate antidiuretic hormone secretion (SIADH). Clinically, a significant number of patients present with hyponatremia as the initial symptom, which can easily lead to misdiagnosis. We report a case involving a 59-year-old male who exhibited neuropsychiatric symptoms and developed severe hyponatremia due to SIADH. Pathological examination confirmed a diagnosis of SCLC. Based on our patient’s case, in patients with severe hyponatremia, it is crucial to consider whether the condition is caused by lung cancer-associated SIADH. Due to the rarity of this condition, clinicians should enhance their awareness to achieve early diagnosis and treatment.

## Case presentation

A 59-year-old man was admitted to our hospital due to generalized weakness for 20 days, predominantly affecting the lower limbs, described as a sensation of walking on cotton. This condition worsened with nausea and vomiting for one day. The patient had a five-year history of hypertension, treated with oral irbesartan (75 mg daily), and diabetes, managed with oral metformin (0.85 mg bid). The patient denied any history of smoking. On physical examination, his pulse rate was 80 beats per minute, blood pressure was 120/80 mmHg, and body temperature was 36.4 °C. Neurologic examination was normal, and lung auscultation was unremarkable.

**Figure 1:**
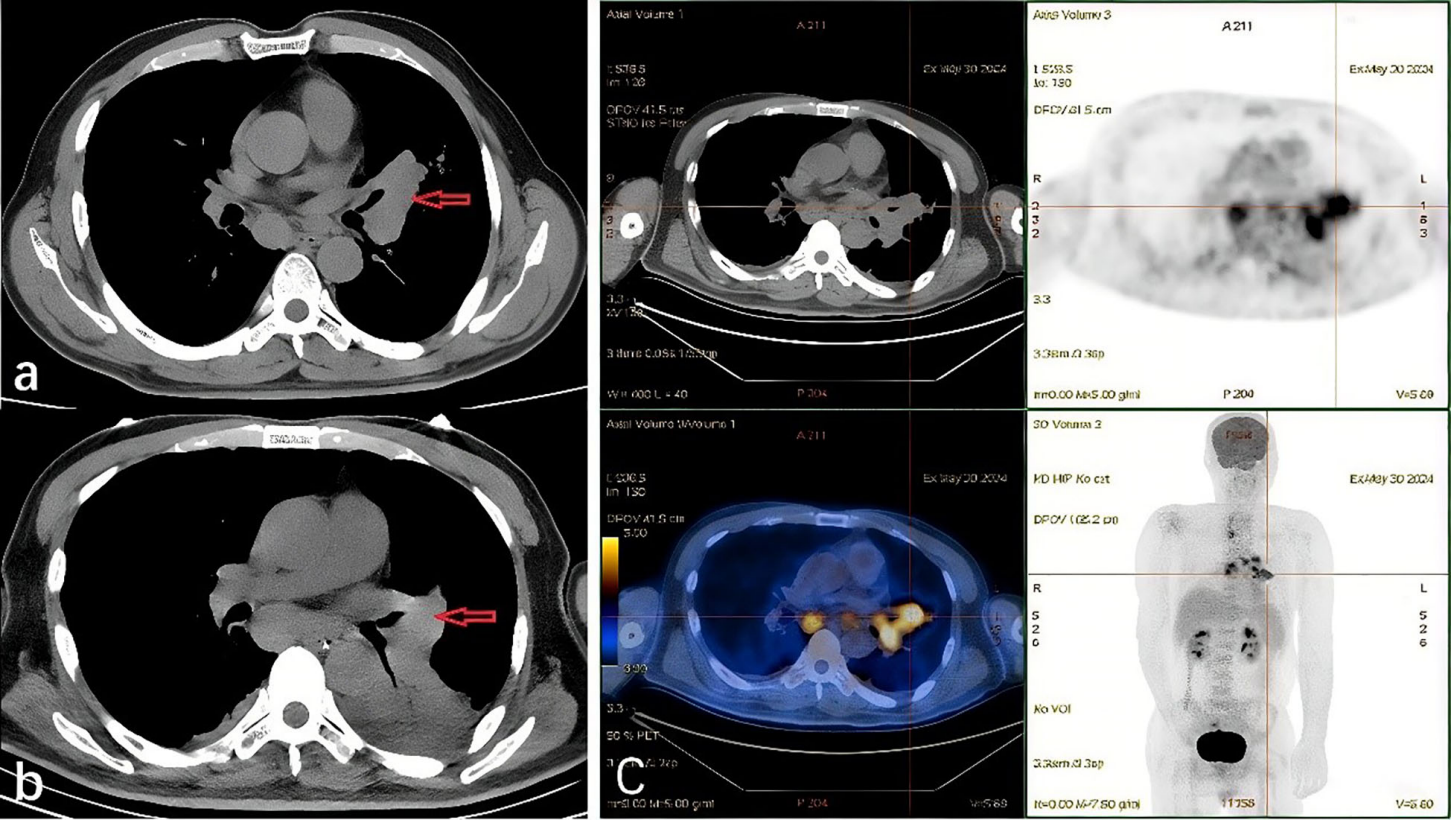
**(a)** Patient’s chest CT after the admission showed the pulmonary consolidation in the left lung, enlarged lymph nodes in the hilum and mediastinum. **(b)** Patient’s chest CT after the treatment showed an increased pulmonary mass and lymph nodes. **(c)** Patient’s Positron Emission Tomography Computed Tomography (PET CT) showed the pulmonary hypermetabolic consolidation (SUVmax=5.69), suggesting malignancy, and there are multiple high metabolic lymph nodes in the mediastinum and pulmonary hilum, indicating metastasis.

A head CT scan revealed multiple lacunar cerebral infarctions. An MRI showed multiple lacunar infarctions, softening foci, leukoaraiosis, and brain atrophy. A chest CT revealed a malignant lesion at the left pulmonary hilum, about 6 cm in diameter, invading the left pulmonary vessels, with multiple enlarged lymph nodes in both pulmonary hilums and the mediastinal space (**Figure 1a**). PET-CT indicated increased FDG metabolism in the mass near the left lung hilum, suggestive of malignancy with surrounding inflammation, and enlarged lymph nodes in the left hilum and mediastinum, suggestive of metastasis (**Figure 1c**).

A blood ion assay revealed sodium at 123 mmol/L and chloride at 88.4 mmol/L. Lung cancer tumor markers showed a significant elevation in neuron-specific enolase level to 36.30 ng/mL. We considered the patient to have severe hyponatremia and initiated sodium supplementation to correct the electrolyte imbalance. Despite treatments, including a high-salt diet and intravenous hypertonic saline supplementation, blood sodium levels did not significantly increase. The patient’s 24-hour urinary sodium concentration was 251.2 mmol/L alongside severe hyponatremia. Some laboratory test results during treatment are shown in **Table 1**. Considering the patient’s condition, a pulmonary neuroendocrine tumor with SIADH was suspected.

**Table 1:** Laboratory results during hospitalization.

Variables	Results	References
FT3	3.81	2.76–6.45 pmol/L
FT4	20.19	11.20–23.81 pmol/L
TSH	1.048	0.35–5.10 mIU/L
Cortisol(8:00)	9.76	6.02–18.40 μg/dL
Cortisol(0:00)	8.76	6.02–18.40 μg/dL
Cortisol(16:00)	7.05	2.68–10.50 μg/dL
ACTH(0:00)	27.7	7.2–63.3 pg/mL
ACTH(8:00)	16.5	7.2–63.3 pg/mL
ACTH(16:00)	8.1	3.6–31.7 pg/mL
FSH	6.0	1.27–19.26 mIU/mL
LH	1.66	1.24–8.62 mIU/mL
Testo	1.2↓	1.75–7.81 ng/mL
PRL	11	2.64–13.13 ng/mL
PRA	0.733	Upright position 0.10 - 6.56; Supine position 0.15 - 2.33 (ng/ml)
ALD	109	Upright position 70 - 300; Supine position 30 - 160 (pg/mL)
AII	53	Upright position 50 - 120; Supine position 25 - 60 (pg/mL)
AI0°C	0.397	ng/mL
AX37°C	1.130	ng/mL
ALD/PRA	14.87	<25 ng/dl:ng/ml.h
NSE	36.3↑	0–16.3 ng/mL
ProGRP	2716.0↑	0–65.7 pg/mL

FT3, Free Triiodothyronine; FT4, Free Thyroxine; TSH, Thyroid Stimulating Hormone; ACTH, Adrenocorticotrophic Hormone; FSH, Follicle Stimulating Hormone; LH, Luteinizing Hormone; PRL, Prolactin; PRA, Plasma Renin Activity; ALD, Aldosterone; AII, Angiotensin II; AI0°C, Angiotensin I at 0°C; AX37°C, Angiotensin I at 37°C; ALD/PRA, Aldosterone/Plasma Renin Activity Ratio; NSE, Neuron-Specific Enolase; ProGRP, Pro-Gastrin-Releasing Peptide.

**Figure 2:**
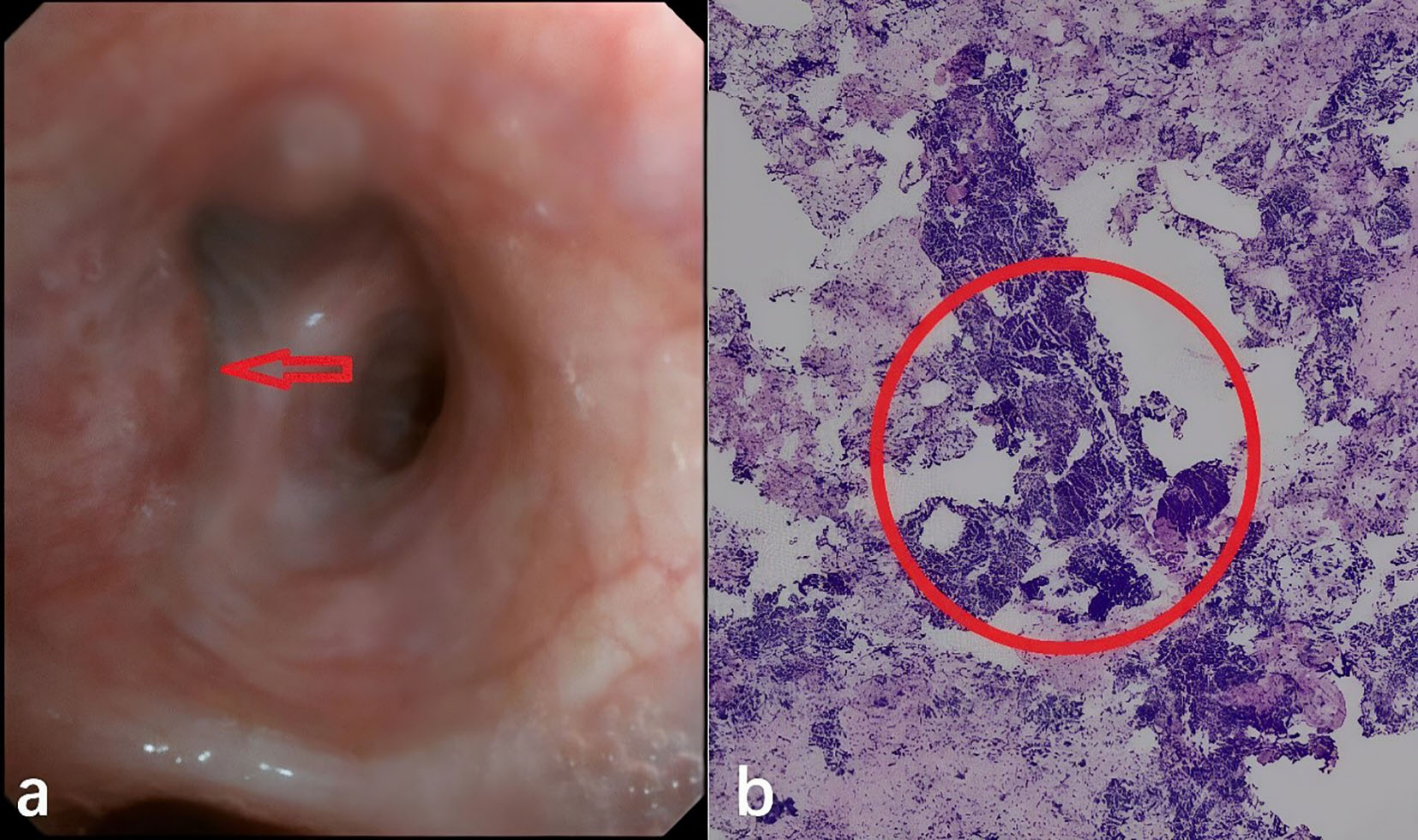
**(a)** Patient’s tracheoscopy picture at the trachea opening in the upper lobe of the left lung, the arrow indicates tumor invasion into the mucosal layer of the bronchus. **(b)** Patient’s pathological slides of seven groups of lymph node puncture biopsies performed by ultrasound tracheoscopy. Within the red circles, it’s shown that small, round, nest-like, and hyperchromatic neuroendocrine tumor cells.

Subsequently, bronchoscopic biopsy confirmed SCLC in the bronchial mucosa and lymph nodes of the left upper lobe (**Figures 2a, b**). Oral tolvaptan (15 mg daily) was administered, normalizing serum sodium to 138.9 mmol/L. Soon after, the patient commenced the first chemotherapy cycle with Etoposide (70 mg daily), Lobaplatin (100 mg daily), and Adebrelimab (1200 mg single treatment). On the third day of chemotherapy, the patient suffered persistent seizures, accompanied by a drop in blood pressure and oxygen saturation, requiring intubation and transfer to the intensive care unit. A blood ion assay indicated sodium at 123 mmol/L compared to 138.9 mmol/L before chemotherapy. The patient’s comorbidities included severe hyponatremia, respiratory failure, acidosis, and hypoproteinemia. Intravenous hyperalimentation, sodium supplementation, and sodium valproate for seizure control and symptomatic treatment were administered.

A lumbar puncture found no tumor cells in cerebrospinal fluid pathology. A head CT scan review showed no new abnormalities. A chest CT scan revealed a slight tumor increase, with bronchial stenosis in the left lower lobe, pulmonary consolidation, and increased lymph node size in the hilar and mediastinal areas compared to previous scans (**Figure 1b**).

**Figure 3:**
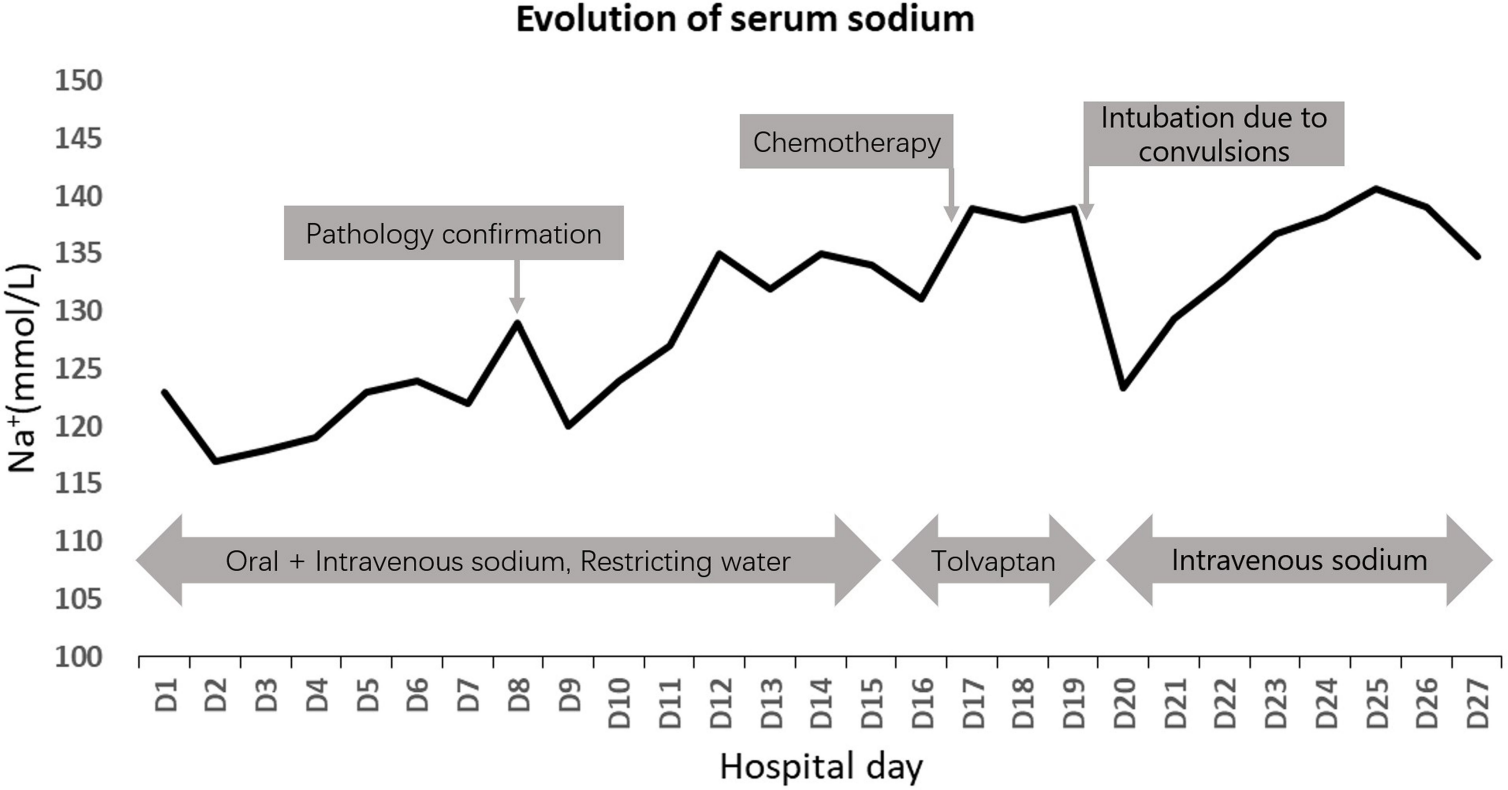
Patient’s evolution of serum sodium during the treatment.

The patient continued to experience unexplained recurrent seizures despite hyponatremia correction and developed multiple complications such as pulmonary infection and respiratory failure. A comprehensive hospital consultation determined the patient was not an eligible candidate for chemotherapy. After family discussions, they decided to discontinue treatment and discharge the patient. Changes in blood sodium levels during treatment are shown in **Figure 3**.

## Literature review

A comprehensive literature search was conducted in PubMed, Scopus, Embase, and the Web of Science (WOS) databases for articles published up to July 2025. The search strategy utilized the following keywords: “small cell lung cancer”, “hyponatremia”, and “syndrome of inappropriate antidiuretic hormone secretion”. Inclusion criteria were defined as follows: (1) Patients diagnosed with SCLC during a medical consultation. (2) Comprehensive clinical date. (3) Article type limited to case reports. The exclusion criteria were as follows: (1) Articles published in languages other than English. (2) Duplicate publications.

**Figure 4:**
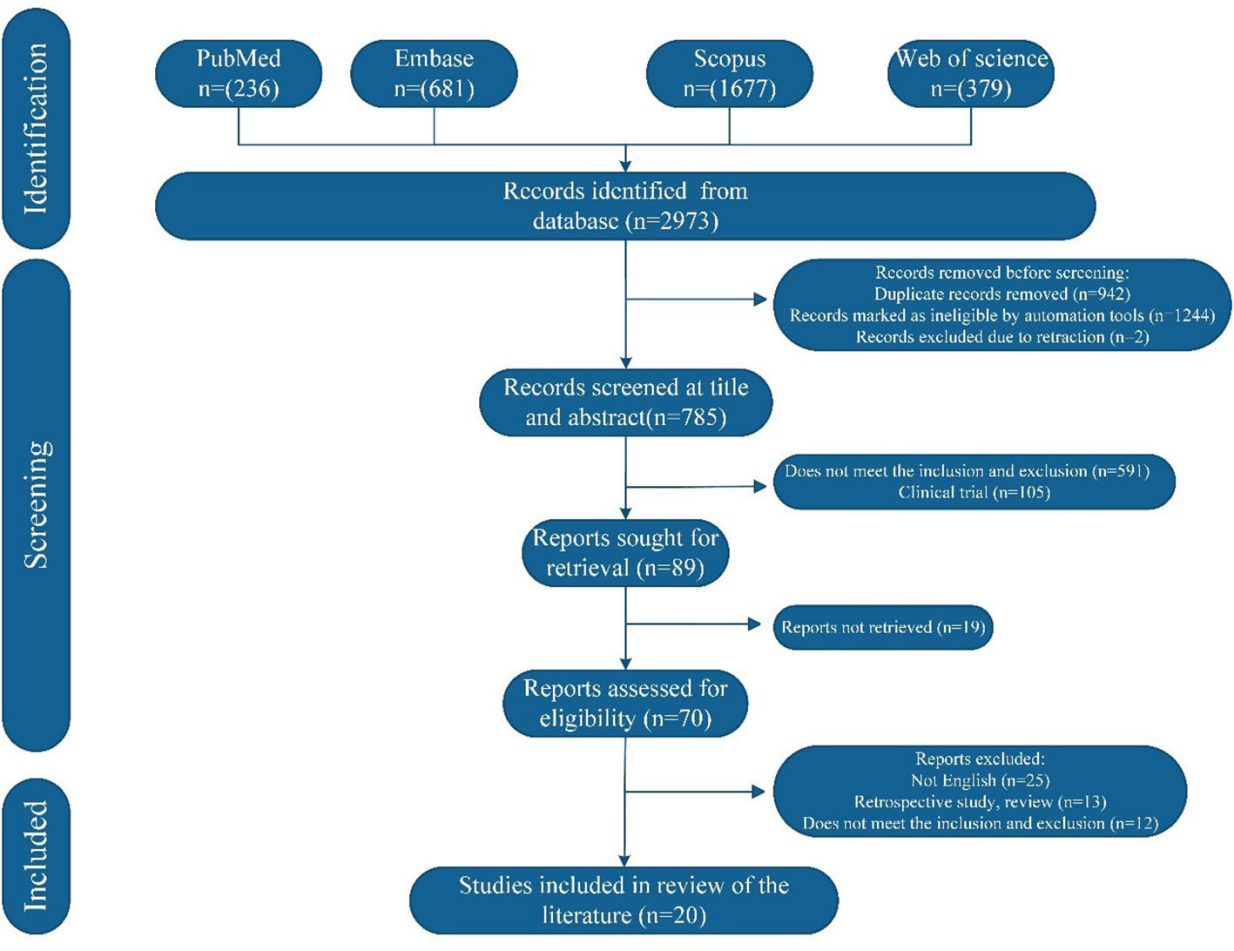
The flowchart of papers identification and inclusion process.

The initial search identified 2973 studies, of which 2188 were excluded before screening. After title and abstract screening, 89 studies were deemed potentially relevant and underwent full-text review. Following eligibility assessment, 20 studies met the inclusion criteria and a total of 23 cases were analyzed. The study selection process is summarized in **Figure 4** and the demographic and clinical characteristics of the included patients are detailed in **Table 2**.

**Table 2:** List of clinical data of 23 cases of small cell lung cancer with hyponatremia.

Study	Cases	Country	Age	Sex	Serum sodium on admission	Relevant clinical information regarding SCLC	Maximum dimension	Presenting symptoms		Comorbidity	Smoking history	Treatment	Outcomes
Taskaldiran et al.; 2023 (1)	1	Turkey	68	Male	113 mmol/L	Organ metastasis (liver)	N/A	Weak and dizzy	N/A		Yes	N/A	Dead in a short time
Tyre et al.; 2023 (2)	2	USA	58	Male	120 mmol/L	Right hilar mass measuring 5 cm x 4 cm, bilateral nodularities in the lower lobes w/bilateral atelectasis, a spiculated nodule in the right lower lobe measuring 2.5 cm x 2 cm, and diffuse emphysematous change	5cm x 4cm	None	N/A		Yes	N/A	N/A
Kai et al.; 2019 (3)	3	Japan	45	Male	113 mmol/L	SCLC (pT2bN3M0)	N/A	Personality disorder and agitation	N/A		Yes	Cisplatin-based chemotherapy; tolvaptan; dietary salt intake; fluid restriction; amino acid supplementation	Dead within approximately 1 year after diagnosis
	4	Japan	88	Male	129 mmol/L	SCLC (pT4N2M1a)	N/A	None	N/A		N/A	Best supportive care; tolvaptan; dietary salt intake; fluid restriction;	Dead
Agarwal et al.; 2018 (4)	5	USA	55	Female	126 mmol/L	Organ metastasis (liver, right femur, and ribs)	N/A	None	N/A		Yes	Palliative chemotherapy (carboplatin; etoposide)	Dead within 2 months after diagnosis
Peri et al.; 2017 (5)	6	Italy	65	Male	117 mmol/L	SCLC-mediastinal-hilar mass in left lung with involvement of the aortopulmonary window and infiltration of trachea and left bronchus; Organ metastasis (lung and liver)	N/A	N/A	Hypertension		N/A	Chemotherapy (Cisplatin; etoposide); tolvaptan	N/A
	7	Spain	68	Female	124 mmol/L	SCLC (T3N3M1b)	N/A	Dizziness and impaired cognitive function	Hypertensive heart disease		N/A	Chemotherapy (Cisplatin; etoposide); tolvaptan	N/A
Kalhan et al.; 2017 (6)	8	UK	68	Female	100 mmol/L	A 4 cm left hilar mass invading the mediastinum with level 4 lymph node enlargement.	4 cm	Weakness, confusion and mild slurring of speech	Type II diabetes mellitus, primary autoimmune hypothyroidism and hypertension		N/A	Chemotherapy	N/A
Altraja et al.; 2015 (7)	9	Estonia	57	Male	104 mmol/L	Extensive stage SCLC (lymph node and liver metastases	N/A	Acute neurological symptoms (mainly disorientation)	N/A		Yes	Chemotherapy (cisplatin, etoposide and topotecan); fludrocortisone	Dead within progressive SCLC approximately 9 months later
Ardizzoni et al.; 2015 (8)	10	Italy	59	Female	113 mmol/L	A right lung hilum tumor mass	N/A	Nausea and dizziness	Hypertension, hyperlipidemia		N/A	Chemotherapy (cisplatin and etoposide); tolvaptan	N/A
Luks et al.; 2014 (9)	11	Washington	68	Female	131 mmol/L	A 4.5-cm mass in the right upper lobe, extensive mediastinal and hilar lymphadenopathy, and an endobronchial soft tissue mass in the carina and right main stem bronchus	4.5cm	Hoarseness, dysphagia, and unintentional weight loss	N/A		Yes	Palliative chemotherapy (cisplatin and etoposide)	N/A
Atkin et al.; 2011 (10)	12	N/A	70	Female	122 mmol/L	A lesion in the left lung	N/A	None	N/A		Yes	Tolvaptan	N/A
Ekamol et al.; 2011 (11)	13	N/A	60	Male	117 mmol/L	A focal irregular density in the left lung apex and mediastinal mass extending to the left hilum. And liver metastases and liver metastases	6.2x4 cm	Nausea, vomiting, and generalized malaise	Type 2 diabetes, hypertension, hyperlipidemia, gout		Yes	Chemotherapy (cisplatinum and irinotecan); fluid restriction; oral demeclocycline	Dead within one and a half months after the diagnosis of SCLC
Alexandrescu et al.; 2010 (12)	14	USA	56	Male	118 mmol/L	Enlarged mediastinal nodes and a left hilar mass; Organ metastasis (brain and liver)	2.7 cm	Confusion, increasing fatigue, weakness, and a 30-pound weight loss	Type 2 diabetes and hypertension,		Yes	Chemotherapy (carboplatin and etoposide); fluid restriction; oral demeclocycline	Dead within 4.5 months after his original diagnosis of extensive stage SCLC
Kneebone et al.; 2009 (13)	15	Australia	73	Female	N/A	A 2 cm mediastinal lymph node	N/A	Progressive leg weakness followed by progressive lower limb sensory disturbance with urinary and fecal incontinence	Chronic airways disease and restless legs syndrome		Yes	N/A	Dead within 6 weeks post-admission
Yang et al.; 2006 (14)	16	Taiwan	59	Male	125 mmol/L	An enlarged mediastinum	N/A	Headache, nausea, retrograde amnesia, altered mental status, and bizarre behavior	N/A		N/A	Chemotherapy (cisplatin and etoposide)	A stable condition during the 6 months of follow-up.
Kaneko et al.; 1998 (15)	17	Japan	72	Male	121 mmol/L	An enlargement of the paraaortic, paratracheal, anterosuperior mediastinal and right hilar lymph nodes	7mm	Asymptomatism	Normocytic anemia;		N/A	Demeclocycline; vincristine; Adriamycin; etoposide and cisplatin combine irradiation; diet of high sodium content;	N/A
Fraser et al.; 1994 (16)	18	UK	72	Male	117 mmol/L	A right hilar tumor with mediastinal lymph node involvement	N/A	Chest pain and weight loss	Myocardial infarction and peptic ulceration		Yes	No	Deteriorated rapidly and died
Pierce et al.; 1992 (17)	19	USA	61	Male	126 mmol/L	A right paratracheal mass; metastatic organ (the hilar lymph nodes, thyroid, right adrenal gland, and pancreas)	2cm	A more aggressive personality and anxiety; hoarseness; weight loss	N/A		Yes	Chemotherapy (etoposide, cisplatin, cyclophosphamide, doxorubicin and vincristine); fluid restriction; demeclocycline; oral potassium	Died on the fourth day in the hospital, 127 days after diagnosis
Kamoi et al; 1987 (18)	20	Japan	62	Male	I17 mmol/L	A mass shadow in the left lung; metastatic organ (both lungs; the left atrial subendocardium)	N/A	Chest pain and a dry cough	No		N/A	Chemotherapy (vincristine, prednisone, endoxan and Adriamycin, etoposide, cisplatin); radiation;	Dead
Suzuki et al.; 1984 (19)	21	Japan	63	Male	126 mmol/L	An abnormal shadow in the right upper lung field and widening of the mediastinum; metastatic organ (liver and bone)	N/A	Productive cough, back pain, dyspnea, anorexia, weight loss and weakness in both legs	N/A		Yes	Chemotherapy (Nimustine hydrochloride)	Dead on the 42nd hospital day
Hirata et al.; 1976 (20)	22	Japan	63	Female	102 mmol/L	A solid, white tumor weighing 670 gm, was present in the right lung hilus; metastatic organ (the anterior mediastinum and the supraclavicular lymph nodes bilaterally)	N/A	Anorexia, vomiting, lethargic and mildly confused	N/A		N/A	Intensive chemotherapy	Dead
	23	Japan	66	Male	112 mmol/L	A large, firm tumor was located in the left hilar region, and metastases to the mediastinum, lymph nodes of the subaortic fossa, and the subphrenic region were noted	N/A	Anorexia and backache	Pulmonary tuberculosis		Yes	A left pneumonectomy a left pneumonectomy; fluid restriction	Dead

N/A indicates data that were not reported, not available, or not applicable in the context of the original publication or clinical record. This may include missing information, unmeasured variables, or instances where the parameter was not relevant to the case.

## Discussion

SCLC accounts for 10%-15% of all primary bronchial lung cancers (21). It predominantly affects the large bronchi and is characterized by poor differentiation, rapid proliferation, high malignancy, and early hematogenous metastasis (22). Paraneoplastic syndromes affect approximately 10% of patients with lung cancer. Among the most frequently observed are humoral hypercalcemia of malignancy (HHM), commonly associated with squamous cell carcinoma, and the SIADH, frequently seen in SCLC (23). SCLC is the most common malignant tumor associated with paraneoplastic syndromes, with hyponatremia being one of the most frequent syndromes due to ectopic hormone secretion. A cohort study in Denmark, which included 6,995 patients with non-small cell lung cancer (NSCLC) and 1,171 patients with SCLC, found that the incidence of hyponatremia was 16% in patients with NSCLC and 26% in patients with SCLC, with a significant association between hyponatremia and low survival rates (24). In NSCLC, the possibility of radical resection can play a pivotal role in resolving paraneoplastic syndromes, particularly when the underlying tumor is the primary driver of systemic manifestations. Surgical removal of the tumor often leads to rapid improvement or complete resolution of symptoms such as hypercalcemia or ectopic hormone production. However, paraneoplastic syndromes associated with SCLC are common but rarely amenable to surgical resolution due to the disease’s aggressive nature and frequent metastatic spread at diagnosis. Instead, systemic therapies—particularly chemotherapy—are typically required to control both the primary tumor and associated paraneoplastic manifestations such as SIADH or ectopic adrenocorticotropic hormone (ACTH) production.

## Causes of hyponatremia

Hyponatremia is common during cancer treatment and related to factors such as primary tumors, brain metastases, pituitary-thyroid-adrenal insufficiency, pneumonia and other lung diseases, SIADH, as well as chemotherapeutic, immunotherapeutic, and targeted drugs. The most severe and malignant cause of hyponatremia is primary malignant tumors, especially SCLC. Ezoe et al. analyzed 29 clinical trial reports and found that the incidence of hyponatremia after platinum-based chemotherapy regimens was 11.9% (25, 26). A case reported by Meena et al. described a 65-year-old patient with NSCLC who developed drowsiness after three weeks of gefitinib treatment and was diagnosed with severe hyponatremia, attributed to gefitinib-induced effects (27). Some studies have reported that immunotherapy may increase the risk of hyponatremia in lung cancer; for example, Nivolumab can induce adrenal insufficiency and hyponatremia (28).

## Syndrome of inappropriate antidiuretic hormone secretion

SIADH refers to excessive secretion or hyperactive function of antidiuretic hormone (ADH) or ADH-like substances, unregulated by negative feedback mechanisms, causing retention of water and sodium, resulting in dilutional hyponatremia. It can occur due to medications, malignant tumors, pulmonary diseases, infections, and central nervous system disorders (29). ADH is synthesized in the hypothalamus and released by the posterior pituitary (neurohypophysis). It plays a central role in maintaining fluid homeostasis by binding to receptors in the kidneys and promoting water reabsorption, thereby reducing free water excretion. Under normal physiological conditions, an increase in plasma osmolality beyond approximately 280 mOsm/kg stimulates ADH secretion, which facilitates water retention in the renal collecting ducts to maintain osmotic equilibrium. In SCLC, tumor cells aberrantly express ADH mRNA and ectopically secrete ADH, disrupting normal feedback control and leading to elevated circulating ADH levels. The resulting impaired free water clearance in the distal nephron contributes to dilutional hyponatremia. Interestingly, not all patients with SCLC-associated hyponatremia exhibit elevated plasma ADH levels. In some cases, tumor cells may instead express atrial natriuretic peptide (ANP) mRNA and secrete ANP, which promotes natriuresis and contributes to hyponatremia via an alternative pathophysiological pathway (30, 31). In 1957, Schwartz et al. identified SIADH as primarily causing hyponatremia in pulmonary malignancies, with approximately 70% of related cases being SCLC. It results from tumor-induced ADH secretion, enhancing water reabsorption (32). Literature indicates SIADH complicating SCLC in 10-16% of cases, and 2-4% in NSCLC{sp} (4, 33, 34){/sp}. SIADH symptoms may precede, coincide with, or follow lung cancer symptoms, often leading to misdiagnosis due to their atypical nature. The diagnosis of SIADH is based on specific laboratory findings, including: 1) Serum sodium <130 mmol/L, plasma osmolality <275 mOsm/kg; 2) Urine osmolality >100 mOsm/kg; 3) Normal blood volume; 4) Urinary sodium concentration >30 mmol/L with normal salt intake; 5) Normal thyroid, adrenal, and kidney function (35).

SIADH represents the most prevalent neuroendocrine paraneoplastic syndromes associated with SCLC, with up to 15% of SCLC exhibiting SIADH, which contributes to a poorer prognosis for SCLC (36). In patients with SCLC, hyponatremia frequently arises from the ectopic secretion of ADH, a hallmark of SIADH in malignancies. Beyond paraneoplastic ADH production, pharmacologic agents used in cancer therapy represent a major contributing factor. These agents may induce or exacerbate hyponatremia by stimulating ADH release, enhancing ADH binding to type 2 vasopressin receptors (AVPR2), or via combined mechanisms (37, 38). Notable offenders include several chemotherapeutic drugs—such as cyclophosphamide, vincristine, vinblastine, melphalan, and cisplatin (which more commonly causes salt-wasting nephropathy). In our study, lobaplatin—a third-generation platinum-based chemotherapeutic agent—and adebrelimab—a monoclonal antibody targeting programmed death-ligand 1 (PD-L1)—were utilized as part of the treatment regimen. Both agents have been associated with an increased risk of hyponatremia. In a pooled analysis of 29 clinical trials, Ezoe et al. reported that the incidence of grade 3/4 hyponatremia was 11.9% among patients receiving platinum-based chemotherapy, significantly higher than those treated with non–platinum-based regimens (25). Moreover, emerging cancer therapies, particularly molecularly targeted agents and immune checkpoint inhibitors (ICIs), warrant careful consideration regarding electrolyte disturbances. A meta-analysis and trial sequential analysis of randomized controlled trials conducted by Moraes et al. demonstrated a significantly higher incidence of hyponatremia—of any grade—in patients receiving PD-1/PD-L1 inhibitors compared to control groups (39, 40). The underlying mechanism may involve ACTH deficiency, which can lead to secondary adrenal insufficiency and, consequently, hyponatremia due to reduced cortisol production. In addition, non-pharmacologic factors commonly encountered in oncology practice may worsen hyponatremia. These include nausea, vomiting, pain, emotional stress, fluid overload related to chemotherapy administration, diarrhea, and comorbid heart or renal failure (41).

## Prognosis of hyponatremia

As a highly malignant tumor, SCLC is typically treated with chemotherapy and radiotherapy. Patients with tumors accompanied by hyponatremia often have poor health; thus, sodium levels are considered a marker of disease severity. A retrospective study analyzing 999 patients with SCLC found that the overall survival (OS) of 226 hyponatremia patients was shorter compared to those with normal sodium levels (11.7 *vs*. 10.0 months, *P*=0.039). The OS of 119 untreated patients with hyponatremia was also shorter (6.8 *vs*. 5.2 months, *P*=0.009) *(* 42). Other studies indicate hyponatremia as an independent adverse prognostic factor, irrespective of other prognostic variables (43). After chemotherapy, hyponatremia may resolve due to drug effects, yet tumor cell destruction might lead to increased ADH secretion, exacerbating hyponatremia. Persistent or recurrent hyponatremia often signals ineffective chemotherapy and disease progression (44).

## Treatment of SIADH

Early detection and correction are essential for improving quality of life and reducing mortality (45). For patients with SCLC who had mild to moderate hyponatremia, daily water intake restriction to 800–1000 mL may provide relief, though progression can be slow. For serum sodium <120 mmol/L, 3% concentrated sodium chloride intravenous infusion, coupled with oral salt capsules, is recommended to increase sodium levels. Emergency treatment with hypertonic saline is warranted if neurological symptoms are present (46). For non-responsive patients, antidiuretic hormone V2 receptor antagonists can facilitate water excretion and correct hyponatremia. Tolvaptan has demonstrated stable corrective effects on hyponatremia in advanced SCLC with SIADH, outperforming diets and fluid restriction (47). Various drugs such as Conivaptan, Tolvaptan, and Mozavaptan are currently in clinical use, with Tolvaptan being widely employed (48, 49). Other treatments include urea, demeclocycline, and fludrocortisone (50, 51).

## In conclusion

In clinical practice, when unexplained refractory hyponatremia coexists with pulmonary lesions, the possibility of pulmonary malignant tumors with SIADH should be considered. Prompt diagnosis based on SIADH criteria is crucial to determine its cause. When correcting hyponatremia, clinicians should limit fluids, supplement sodium ions, and possibly use antidiuretic hormone antagonists to improve prognosis. Hyponatremia during chemotherapy requires careful management. More clinical research is needed to ascertain whether hyponatremia independently affects the prognosis of patients with SCLC.

## Data Availability

The original contributions presented in the study are included in the article/supplementary material. Further inquiries can be directed to the corresponding author.
